# Association of Sleep Duration with Obesity and Cardiometabolic Risk Factors in Children and Adolescents: A Population-Based Study

**DOI:** 10.1038/s41598-019-45951-0

**Published:** 2019-07-01

**Authors:** Sook Hyun Seo, Young Suk Shim

**Affiliations:** Department of Pediatrics, Hallym University Dongtan Sacred Heart Hospital, Hwaseong, Korea

**Keywords:** Obesity, Metabolic syndrome

## Abstract

This study aimed to evaluate the relationships between sleep duration and overweight/obesity, obesity, and metabolic syndrome (MetS) and its components in children and adolescents. A total of 6,048 participants aged 10–18 years were divided into the following four sleep-duration groups based on age-specific sleep duration: i) very short; ii) short; iii) recommended; and iv) long. The participants in the very short sleep-duration group had an increased odds ratio (OR) of 1.76 for overweight/obesity, 1.69 for obesity, and 1.49 for elevated waist circumference (WC) compared with the recommended sleep-duration group. The subjects in the long sleep-duration group had an increased OR of 2.71 for elevated triglyceride (TG) compared with those in the recommended sleep-duration group. In subgroup analyses, boys in the very short sleep-duration group exhibited an increased OR of 1.78 for overweight/obesity compared with those in the recommended sleep-duration group. Compared with girls in the recommended sleep-duration group, those in the very short sleep-duration group exhibited an increased OR of 1.69 for overweight/obesity, 2.28 for obesity, and 1.57 for elevated WC; in contrast, girls in the very short sleep-duration group exhibited a decreased OR of 0.58 for elevated TG. The girls in the long sleep-duration group had an increased OR of 3.86 for elevated TG compared with those with recommended sleep-duration. Our results suggest that shorter sleep durations may be related to overweight/obesity, obesity, and central obesity, and longer sleep durations may be associated with elevated TG. However, the nature of these relationships may be dependent on sex.

## Introduction

Childhood and adolescent overweight/obesity have become a major concern for public health worldwide^1^. With the increasing prevalence of childhood and adolescent obesity, the prevalence of metabolic syndrome (MetS), which is a clustering of abdominal obesity (elevated waist circumference (WC)), elevated blood pressure (BP), elevated glucose, and dyslipidemia (elevated triglyceride (TG) or reduced high-density lipoprotein cholesterol (HDL-C)), has also increased. Overweight/obesity in children and adolescents is often related to obesity in adults^2^ and obesity-related complications in adults, including hypertension^3^, type 2 diabetes mellitus (T2DM)^4^, dyslipidemia^3^, and MetS^5^. Because the constellation of cardiometabolic risk factors is considered to be modifiable, the early identification of and intervention for children and adolescents with MetS is an important contention for public health to improve their condition.

Sleep is considered to play a role in growth and development in children and adolescents and in health status in children, adolescents, and adults through its control over the diurnal rhythm, which is related to energy homeostasis^6^. Recent evidence from epidemiological studies has indicated that an increasing prevalence of obesity is related to decreased sleep duration in both children and adults^1,7^. In adult studies, short sleep duration may be a risk factor for the development of obesity^8^, diabetes^9^, and cardiovascular disease^10^. However, some studies have reported differences in the relationships between impaired sleep duration and obesity and cardiometabolic risk factors based on age^9^ and sex^11^. A few studies have shown that short sleep duration is related to childhood obesity^12,13^, although little research suggested that decreased sleep duration is related to cardiometabolic risk factors in the childhood period^14^. In general, studies regarding the relationships between impaired sleep duration in adolescents and obesity and cardiometabolic risk factors have been limited.

In the present population-based study, we aimed to evaluate the relationships between sleep duration and overweight/obesity, obesity, and MetS and its components and to investigate whether these relationships persisted after adjustment for possible confounders in children and adolescents aged 10–18 years using nationally representative data. Additionally, we examined these relationships based on sex differences.

## Results

### Clinical characteristics of the study population in the sleep-duration groups according to sex

The clinical characteristics of the study population are shown in Table 1. Boys in the very short sleep-duration group had a higher mean age (*P* < 0.001), weight standard deviation score (SDS) (*P* < 0.001), body mass index (BMI) SDS (*P* < 0.001), WC SDS (*P* < 0.001), and diastolic BP (DBP) *P* = 0.033), but lower mean glucose (*P* < 0.001) and HDL-C (*P* = 0.033) levels. Boys in the long sleep-duration group were more likely to be alcohol drinkers (*P* = 0.001), smokers (*P* < 0.001), and rural residents (*P* = 0.015). Girls in the very short sleep-duration group had a higher mean age (*P* < 0.001), weight SDS (*P* < 0.001), BMI SDS (*P* < 0.001), systolic BP (SBP) (*P* = 0.007), and DBP (*P* = 0.004), whereas girls in the long sleep-duration group had higher mean TG (*P* < 0.001) levels. Girls in the long sleep-duration group were more likely to be smokers (*P* < 0.001) and rural residents (*P* = 0.008), whereas girls in the very short sleep-duration group were more likely to be alcohol drinkers (*P* = 0.003).

**Table 1:** Clinical characteristics of the study population (*n* = 6,048).

	Sleep duration				*P*
	Very short	Short	Recommended	Long	
**Boys**	*n* = 349	*n* = 1,937	*n* = 914	*n* = 18	
Age (years)	15.84 ± 1.83	14.34 ± 2.13	14.64 ± 1.93	14.56 ± 1.50	<0.001
Height SDS	0.65 ± 1.05	0.81 ± 1.05	0.64 ± 1.03	0.95 ± 1.09	0.102
Weight SDS	0.48 ± 1.02	0.46 ± 1.03	0.23 ± 1.07	0.44 ± 0.89	<0.001
BMI SDS	0.21 ± 1.10	0.12 ± 1.06	−0.07 ± 1.09	0.01 ± 0.89	<0.001
WC SDS	−0.03 ± 1.17	−0.15 ± 1.11	−0.33 ± 1.16	−0.41 ± 0.95	<0.001
SBP (mmHg)	110.61 ± 9.70	109.37 ± 10.79	109.32 ± 10.54	111.39 ± 11.03	0.193
DBP (mmHg)	69.93 ± 8.16	67.15 ± 9.54	67.74 ± 9.41	67.33 ± 8.30	0.033
Glucose (mg/dL)	88.43 ± 6.93	90.21 ± 6.45	90.36 ± 6.68	89.06 ± 3.61	<0.001
T-C (mg/dL)	152.40 ± 27.47	151.88 ± 26.55	151.84 ± 26.45	152.33 ± 28.53	0.801
HDL-C (mg/dL)	47.07 ± 9.52	48.69 ± 9.23	48.68 ± 9.22	49.61 ± 9.15	0.033
TG (mg/dL)	89.30 ± 53.40	83.71 ± 48.59	84.28 ± 47.26	80.83 ± 35.57	0.244
LDL-C (mg/dL)	87.45 ± 23.99	86.45 ± 23.31	86.30 ± 22.15	86.56 ± 24.56	0.519
Alcohol consumption (%)	34 (9.7%)	102 (5.3%)	85 (9.3%)	3 (16.7%)	<0.001
Smoking (%)	79 (22.6%)	363 (18.7%)	214 (23.4%)	9 (50.0%)	<0.001
Physical activity (%)	43 (12.3%)	245 (12.7%)	110 (12.0%)	2 (11.1%)	0.969
Household income ≤ 1st quartile (%)	35 (10.0%)	213 (11.0%)	124 (13.6%)	2 (11.1%)	0.174
Rural residence (%)	51 (14.6%)	322 (16.6%)	191 (20.9%)	4 (22.2%)	0.015
Hypertension (%)	0 (0%)	0 (0%)	0 (0%)	0 (0%)	>0.999
Diabetes mellitus (%)	0 (0%)	2 (0.10%)	0 (0%)	0 (0%)	0.724
Dyslipidemia (%)	0 (0%)	0 (0%)	0 (0%)	0 (0%)	>0.999
Sleep duration (hr)	4.98 ± 0.62	6.99 ± 0.71	8.73 ± 0.78	11.22 ± 0.43	<0.001
**Girls**	*n* = 428	*n* = 1,674	*n* = 709	*n* = 19	
Age (years)	15.52 ± 1.86	14.38 ± 2.13	14.77 ± 1.99	13.74 ± 1.41	<0.001
Height SDS	0.37 ± 1.02	0.46 ± 1.02	0.37 ± 1.06	0.13 ± 0.89	0.507
Weight SDS	0.37 ± 1.13	0.26 ± 1.00	0.12 ± 1.13	−0.06 ± 1.15	<0.001
BMI SDS	0.27 ± 1.16	0.10 ± 1.00	−0.02 ± 1.12	−0.12 ± 1.23	<0.001
WC SDS	−0.04 ± 1.17	−0.11 ± 1.05	−0.17 ± 1.12	0.00 ± 1.05	0.060
SBP (mmHg)	104.60 ± 9.35	103.86 ± 9.07	103.29 ± 9.32	100.11 ± 10.03	0.007
DBP (mmHg)	67.62 ± 7.98	65.89 ± 7.91	66.01 ± 8.26	64.47 ± 7.41	0.004
Glucose (mg/dL)	87.85 ± 6.89	88.76 ± 6.97	88.74 ± 6.44	89.74 ± 7.30	0.051
T-C (mg/dL)	161.80 ± 28.26	161.05 ± 25.54	161.68 ± 24.48	164.53 ± 25.20	0.857
HDL-C (mg/dL)	52.58 ± 9.92	51.69 ± 9.71	51.73 ± 9.62	47.84 ± 6.48	0.109
TG (mg/dL)	81.10 ± 41.19	84.03 ± 42.27	86.84 ± 41.97	125.84 ± 73.30	0.001
LDL-C (mg/dL)	92.95 ± 24.03	92.54 ± 22.03	92.60 ± 21.63	91.42 ± 22.32	0.793
Alcohol consumption (%)	17 (3.97%)	64 (3.82%)	52 (7.33%)	1 (5.26%)	0.003
Smoking (%)	53 (12.38%)	116 (6.93%)	86 (12.13%)	1 (5.26%)	<0.001
Physical activity (%)	61 (14.25%)	180 (10.75%)	54 (7.62%)	1 (5.26%)	0.004
Household income (%)	44 (10.28%)	172 (10.27%)	101 (14.25%)	2 (10.53%)	0.039
Residence (rural)	54 (12.62%)	277 (16.55%)	141 (19.89%)	4 (21.05%)	0.015
Hypertension (%)	0 (0%)	0 (0%)	0 (0%)	0 (0%)	>0.999
Diabetes mellitus (%)	0 (0%)	0 (0%)	1(0.1%)	0 (0%)	0.393
Dyslipidemia (%)	0 (0%)	0 (0%)	0 (0%)	0 (0%)	>0.999
Sleep duration (hr)	4.93 ± 0.71	6.94 ± 0.75	8.76 ± 0.81	11.42 ± 0.51	<0.001

Data are expressed as the means ± standard deviations (SD).

SDS, standard deviation score; WC, waist circumference; BMI, body mass index; SBP, systolic blood pressure; DBP, diastolic blood pressure; T-C, total cholesterol; TG, triglyceride; HDL-C, high-density lipoprotein cholesterol; LDL-C, low-density lipoprotein cholesterol.

### Adjusted means of cardiometabolic risk factors in the sleep-duration groups according to sex and age

The adjusted means of cardiometabolic risk factors, including BMI SDS, WC SDS, SBP, DBP, and levels of glucose, total cholesterol (T-C), HDL-C, TG, and low-density lipoprotein cholesterol (LDL-C), are provided in Table 2. The boys in the very short sleep-duration group had higher mean BMI SDS (*P* < 0.001 for trend) and WC SDS (*P* < 0.001 for trend), whereas they exhibited lower mean glucose levels (*P* = 0.006 for trend) after adjustment for possible confounders. The very short sleep-duration group of girls had higher mean BMI SDS (*P* < 0.001 for trend) and HDL-C levels (*P* = 0.031 for trend), whereas they exhibited higher TG (*P* < 0.001 for trend) levels. The girls with shorter sleep durations tended to have a higher mean WC SDS (*P* = 0.099 for trend) and DBP (*P* = 0.085 for trend), although the differences were not significant.

**Table 2:** Adjusted means of cardiometabolic risk factors in the sex-specific sleep-duration groups of Korean children and adolescents aged 10–18 years (*n* = 6,048).

	Sleep duration				*P* for trend
	Very short	Short	Recommended	Long	
**Boys**	*n* = 349	*n* = 1,937	*n* = 914	*n* = 18	
BMI SDS^1^	0.24 ± 0.08	0.12 ± 0.06	−0.06 ± 0.06	−0.01 ± 0.26	<0.001
WC SDS^1^	−0.02 ± 0.08	−0.14 ± 0.06	−0.32 ± 0.06	−0.43 ± 0.27	<0.001
SBP^2^	108.28 ± 0.70	109.42 ± 0.49	109.64 ± 0.53	112.14 ± 2.31	0.093
DBP^2^	66.69 ± 0.64	66.17 ± 0.45	66.58 ± 0.48	66.54 ± 2.12	0.595
Glucose^2^	89.47 ± 0.46	90.46 ± 0.33	90.89 ± 0.35	89.61 ± 1.52	0.006
T-C^2^	151.46 ± 1.88	150.48 ± 1.32	151.81 ± 1.41	152.66 ± 6.17	0.608
HDL-C^2^	49.45 ± 0.64	49.99 ± 0.45	49.70 ± 0.48	50.75 ± 2.12	0.663
TG^2^	83.77 ± 3.39	81.13 ± 2.39	83.73 ± 2.54	79.70 ± 11.15	0.497
LDL-C^2^	85.27 ± 1.62	84.29 ± 1.14	85.39 ± 1.22	85.99 ± 5.33	0.620
**Girls**	*n* = 428	*n* = 1,674	*n* = 709	*n* = 19	
BMI SDS^1^	0.40 ± 0.08	0.19 ± 0.07	0.08 ± 0.07	−0.04 ± 0.25	<0.001
WC SDS^1^	0.08 ± 0.08	−0.03 ± 0.07	−0.09 ± 0.07	0.07 ± 0.26	0.099
SBP^2^	104.50 ± 0.70	104.31 ± 0.57	103.90 ± 0.61	101.12 ± 2.12	0.295
DBP^2^	67.45 ± 0.62	66.37 ± 0.50	66.39 ± 0.54	65.41 ± 1.88	0.085
Glucose^2^	88.98 ± 0.52	89.33 ± 0.42	89.60 ± 0.45	90.09 ± 1.58	0.475
T-C^2^	161.18 ± 1.99	160.95 ± 1.62	161.90 ± 1.72	165.04 ± 6.05	0.779
HDL-C^2^	54.08 ± 0.74	52.98 ± 0.60	52.84 ± 0.64	48.86 ± 2.23	0.031
TG^2^	83.15 ± 3.23	84.80 ± 2.63	88.78 ± 2.79	126.48 ± 9.79	<0.001
LDL-C^2^	90.43 ± 1.72	90.99 ± 1.40	91.33 ± 1.48	90.79 ± 5.20	0.933

Data are presented as the estimated means ± SE (standard errors).

SDS, standard deviation score; WC, waist circumference; BMI, body mass index; SBP, systolic blood pressure; DBP, diastolic blood pressure; T-C, total cholesterol; TG, triglyceride; HDL-C, high-density lipoprotein cholesterol; LDL-C, low-density lipoprotein cholesterol.

^1^Models were generated using analysis of covariance (ANCOVA) after adjustment for age, alcohol drinking, smoking, physical activity, household income, and residence according to sex.

^2^Models were generated using analysis of covariance (ANCOVA) after adjustment for age, body mass index (BMI) standard deviation scores (SDS), alcohol consumption, smoking, physical activity, household income, and residence according to sex.

### Adjusted OR of overweight/obesity, obesity, and MetS and its components for the sleep-duration groups according to sex and age

**Figure 1:**
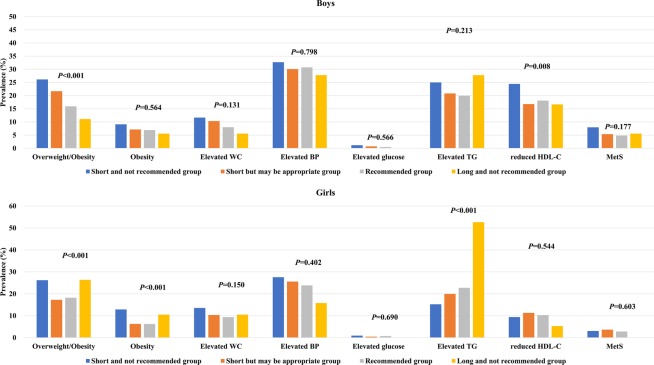
The prevalence of overweight/obesity, obesity, and MetS and its components in sex-specific sleep-duration groups (*n* = 6,048). Statistical significance was determined using chi-square tests.

The prevalence of overweight/obesity, obesity, and MetS and its components is shown in Fig. 1. The overall prevalence of overweight/obesity, obesity, and MetS was 20.5%, 7.3%, and 5.5% in boys and 18.9%, 7.3%, and 3.3% in girls, respectively. The prevalence of overweight/obesity in boys was 25.5% for the very short sleep-duration group, 21.7% for the short sleep-duration group, 16.2% for the recommended sleep-duration group, and 11.1% for the long sleep-duration groups (*P* < 0.001). In girls, the prevalence of overweight/obesity was 26.2% for the very short sleep-duration group, 17.3% for the short sleep-duration group, 18.2% for the recommended sleep-duration group, and 26.3% for the long sleep-duration group (*P* < 0.001). The prevalence of obesity in boys according to sleep duration was 8.9% in those in the very short sleep-duration group, 7.1% for the short sleep-duration group, 7.0% for the recommended sleep-duration group, and 5.6% for the long sleep-duration groups (*P* = 0.663). In girls, the prevalence of obesity was 12.9% for the very short sleep-duration group, 6.3% for the short sleep-duration group, 6.2% for the recommended sleep-duration group, and 10.5% for the long sleep-duration group (*P* < 0.001). The prevalence of MetS did not significantly vary according to sleep duration in boys (*P* = 0.261) or girls (*P* = 0.603).

The adjusted OR for overweight/obesity, obesity, and MetS and its components is presented in Table 3. Compared with those in the recommended sleep-duration group, the subjects in the very short sleep-duration group exhibited an increased OR of 1.76 (95% CI, 1.43–2.18) for overweight/obesity, 1.69 (95% CI, 1.25–2.29) for obesity, and 1.49 (95% CI, 1.13–1.97) for elevated WC. Compared with those with recommended sleep-duration group, the participants in the short sleep-duration group exhibited an increased OR of 1.18 (95% CI, 1.01–1.38) for overweight/obesity and 1.25 (95% CI, 1.01–1.53) for elevated WC. Compared with those in the recommended sleep-duration group, children and adolescents in the long sleep-duration group had an increased OR of 2.71 (95% CI, 1.36–5.38) for elevated TG.

**Table 3:** Adjusted ORs (95% CI) between sleep duration and obesity, overweight/obesity, and cardiometabolic risk factors in Korean children and adolescents aged 10–18 years (*n* = 6,048).

	Sleep duration			
	Very short	Short	Recommended	Long
**All participants**	*n* = 777	*n* = 3,611	*n* = 1,623	*n* = 37
Overweight/obesity^1^	1.76 (1.43–2.18)	1.18 (1.01–1.38)	Reference	1.12 (0.49–1.02)
Obesity^1^	1.69 (1.25–2.29)	1.04 (0.82–1.32)	Reference	1.28 (0.38–4.23)
Elevated WC ^1^	1.49 (1.13–1.97)	1.25 (1.01–1.53)	Reference	0.94 (0.28–3.09)
Elevated BP ^2^	1.00 (0.83–1.22)	1.01 (0.89–1.16)	Reference	0.78 (0.35–1.72)
Elevated glucose^2^	1.45 (0.54–3.90)	1.09 (0.50–2.39)	Reference	—
Reduced HDL-C^2^	0.96 (0.75–1.24)	0.91 (0.77–1.08)	Reference	0.76 (0.26–2.22)
Elevated TG^2^	0.81 (0.64–1.01)	0.89 (0.76–1.03)	Reference	2.71 (1.36–5.38)
MetS^2^	0.74 (0.47–1.18)	1.07 (0.77–1.48)	Reference	0.83 (0.10–6.80)
**Boys**	*n* = 349	*n* = 1,937	*n* = 914	*n* = 18
Overweight/obesity^3^	1.78 (1.31–2.41)	1.42 (1.15–1.75)	Reference	0.63 (0.14–2.79)
Obesity^3^	1.20 (0.76–1.90)	1.02 (0.76–1.41)	Reference	0.76 (0.10–5.96)
Elevated WC^3^	1.40 (0.93–2.12)	1.33 (1.10–1.76)	Reference	0.63 (0.08–4.82)
Elevated BP^4^	0.81 (0.62–1.07)	0.96 (0.80–1.14)	Reference	0.95 (0.33–2.72)
Elevated glucose^4^	1.78 (0.43–7.43)	1.62 (0.53–4.98)	Reference	—
Reduced HDL-C^4^	1.15 (0.84–1.58)	0.82 (0.67–1.02)	Reference	0.90 (0.25–3.22)
Elevated TG^4^	1.10 (0.80–1.49)	0.95 (0.77–1.16)	Reference	1.61 (0.55–4.70)
MetS^4^	1.02 (0.58–1.81)	0.94 (0.62–1.40)	Reference	1.69 (0.18–15.77)
**Girls**	*n* = 428	*n* = 1,674	*n* = 709	*n* = 19
Overweight/obesity^3^	1.69 (1.26–2.26)	0.94 (0.74–1.18)	Reference	1.55 (0.55–4.40)
Obesity^3^	2.28 (1.49–3.49)	1.05 (0.73–1.52)	Reference	1.85 (0.41–8.32)
Elevated WC^3^	1.57 (1.07–2.30)	1.16 (0.86–1.56)	Reference	1.17 (0.26–5.18)
Elevated BP^4^	1.18 (0.89–1.56)	1.07 (0.87–1.31)	Reference	0.59 (0.17–2.07)
Elevated glucose^4^	1.19 (0.31–4.65)	0.67 (0.22–2.08)	Reference	—
Reduced HDL-C^4^	0.77 (0.50–1.18)	1.09 (0.81–1.46)	Reference	0.51 (0.07–3.97)
Elevated TG^4^	0.58 (0.42–0.81)	0.80 (0.64–1.00)	Reference	3.86 (1.50–9.90)
MetS^4^	0.51 (0.22–1.16)	1.42 (0.80–2.52)	Reference	—

WC, waist circumference; BP, blood pressure; HDL-C, high-density lipoprotein cholesterol; TG, triglyceride; MetS, metabolic syndrome.

^1^Multiple logistic regression models were generated after adjustment for sex, age, alcohol drinking, smoking, physical activity, household income, and residence.

^2^Multiple logistic regression models were generated after adjustment for sex, age, body mass index (BMI) standard deviation scores (SDS), alcohol drinking, smoking, physical activity, household income, and residence.

^3^Multiple logistic regression models were generated after adjustment for age, alcohol drinking, smoking, physical activity, household income, and residence according to sex.

^4^Multiple logistic regression models were generated after adjustment for age, body mass index (BMI) standard deviation scores (SDS), alcohol drinking, smoking, physical activity, household income, and residence according to sex.

In the subgroup analyses, compared with those in the recommended sleep-duration group, boys in the very short sleep-duration group exhibited an increased OR of 1.78 (95% CI, 1.31–2.41) for overweight/obesity. Boys in the short sleep-duration group exhibited an increased OR of 1.42 (95% CI, 1.15–1.75) for overweight/obesity and 1.33 (95% CI, 1.10–1.76) for elevated WC compared with those in the recommended sleep-duration group. Compared with those in the recommended sleep-duration group, girls in the very short sleep-duration group exhibited an increased OR of 1.69 (95% CI, 1.26–2.26) for overweight/obesity, 2.28 (95% CI, 1.49–3.49) for obesity and 1.57 (95% CI, 1.07–2.30) for elevated WC, whereas these girls exhibited a decreased OR of 0.58 (95% CI, 0.42–0.81) for elevated TG. Compared with girls in the recommended sleep-duration group, the girls with long sleep-duration group had an increased OR of 3.86 (95% CI, 1.50–9.90) for elevated TG.

## Discussion

The present population-based study showed that boys and girls in the very short sleep-duration group exhibited higher adjusted means of BMI SDS, and boys in the very short sleep-duration group exhibited higher adjusted means of WC SDS. Girls in the long sleep-duration group had higher adjusted means of glucose and TG levels assessed by ANCOVA after controlling for possible confounders. In a multiple logistic regression analysis, compared with those in the recommended sleep-duration group, children and adolescents in the very short sleep-duration group were at an increased risk for overweight/obesity, obesity, and elevated WC and at a decreased risk for elevated TG after controlling for possible confounders. Compared with those in the recommended sleep-duration group, children and adolescents in the long sleep-duration group had an increased risk for elevated TG in multiple logistic regression analyses. However, the nature of these relationships was sex-dependent. Girls had significant relationships of very short sleep duration with overweight/obesity, obesity, elevated WC, and elevated TG, whereas boys exhibited significant associations of very short sleep duration with overweight/obesity but not among obesity, elevated WC, and elevated TG.

A series of studies has reported the association of sleep duration with overweight and/or obesity in children. Most previous cross-sectional studies showed a negative relationship between sleep duration and overweight/obesity and obesity in children. However, a study from New Zealand^15^ and a one from the U.S.A.^16^, which measured sleep duration objectively using actigraphy, found that compared with longer sleep, shorter sleep was related to obesity. In addition to cross-sectional studies, longitudinal studies showed similar findings. Longitudinal studies from the U.K. and the U.S.A. with relatively large study populations showed a negative correlation between sleep duration and BMI^17,18^. However, differences were suggested in the results regarding the association between sleep duration and obesity. Sleep duration has been related to overweight, but the association was not linear^19^. In a study by Knutson *et al*.^19^, children and adolescents with sleep durations of 7.1–9.0 hours had a significantly increased OR for overweight/obesity, whereas those with sleep durations of 0.5–7.0 hours did not have a significantly increased OR. On the other hand, some studies have suggested sex differences in the relationship between sleep duration and obesity. A study using data from the National Longitudinal Study of Adolescent Health suggested that sleep duration was weakly related to overweight/obesity in boys (adjusted OR = 0.90, *P* = 0.04) but not in girls (adjusted OR = 1.06, *P* = 0.27)^20^. In the present study, boys and girls in the very short sleep-duration group had a higher adjusted mean of BMI SDS using ANCOVA based on sex- and age-specific sleep-duration groups, and all participants exhibited an increased OR for overweight/obesity and obesity based on sleep duration. Girls had an increased OR for overweight/obesity and obesity, but boys had an increased OR only for overweight/obesity. Our findings are in line with the results of previous study regarding negative relationships between sleep duration and obesity^15–18^. However, these associations were inconsistent according to sex in a previous study^20^ and our study. Future studies should evaluate discordant sex-different results.

Possible mechanisms for the link between sleep duration and obesity have been suggested. One possibility is that the link between short sleep duration and obesity, along with the subsequent related diseases, may be related to an increase in appetite^21^. Sleep restriction may play a role in a reduced or loss of inhibition of orexigenic activity in the hypothalamic area, and the decreased inhibition of hypothalamic activity that modulates appetite regulation may lead to increased hunger^22^. Increased hunger may result in not only weight gain as a short-term effect but also obesity as a long-term effect^7^. Another possible pathway is related to hormonal disturbances, including the increased release of growth hormone (GH)^23^ and ghrelin in the daytime^24^ and the increased release of cortisol in the evening^25^. A study of 11 healthy, young nonobese participants with six days of sleep restriction revealed that an altered (biphasic) pattern of GH secretion during sleep restriction led to a longer duration of peripheral exposure to increased GH levels^23^. A study from the U.S.A. regarding sleep duration and changes in hormone levels demonstrated that the sleep debt associated with 4 hours of sleep per night for 6 nights resulted in harmful and elevated evening cortisol levels^25^. In addition, a study of 1,024 adults from the U.S.A. suggested that short sleep duration positively correlated with leptin and negatively correlated with ghrelin, and changes in leptin and ghrelin levels may increase appetite, which could be related to increased BMI^24^. These hormonal changes may be related to obesity (especially central obesity)^23–25^. In the current study, children and adolescents in the very short sleep-duration group exhibited an increased OR for elevated WC in multiple logistic regression analysis among all participants. Our findings support previous laboratory research.

With regard to the relationship between sleep duration and cardiovascular disease such as hypertension^10^, coronary vascular disease^26^, and even mortality^27^, most studies have reported a U-shaped association in the adult population. However, the findings regarding the relationship of sleep duration to MetS, which has been studied in the adult population, are inconsistent^28–31^. Some studies have found a U-shaped relationship between sleep duration and MetS^28,29^. A few studies have demonstrated that only short sleep durations were related to MetS^30,31^. However, other studies have demonstrated an association of MetS with long sleep durations but not short sleep durations^32^. Similar to the results from adult studies, reports regarding the relationships between sleep duration and MetS in children and adolescents have been conflicting. An Iranian study found that short sleep duration was associated with an increased risk of MetS and high BP in children and adolescents^33^. In a recent Chinese study, 238 boys with sleep duration <9 hours were at higher risk for hypertension^34^. In addition, a series of studies suggested a significant association between short sleep duration and cardiometabolic risk factors in children^35,36^. However, studies have not reported the association of sleep duration with MetS. A study from the U.S.A. of 133 obese adolescents aged 10–16.9 years showed that sleep duration was not related to obesity, insulin resistance, or MetS^37^. A Brazilian study showed that decreases in sleep duration were not associated with increases in WC^38^. These discrepant findings may result from differences in ethnicity, age, and sex. Although sleep duration in the present study was not related to MetS, future longitudinal studies should be conducted to provide a more comprehensive evaluation of the relationship between sleep duration and cardiometabolic risk in children and adolescents.

Our study has several possible limitations. First, the present study analyzed data that were cross-sectional in nature; therefore, causality between sleep duration and obesity and cardiometabolic risk factors could not be determined. Second, the sleep duration data in the current study were obtained through a subjective, self-reported method, namely, the following question was asked: “For how long do you usually sleep”? We could not perform the statistical analysis regarding the differences between weekday and weekend sleep. Additionally, we could not analyze the data for sleep quality because the KNHANES did not provide data regarding sleep quality, including sleep apnea, which is considered to play an important role in developing obesity^39^. Third, we did not find a relationship between short sleep duration and MetS and its components in this study. A recent Korean study showed that some variables, including short sleep duration, were linked to elevated BP, although short sleep duration was not associated with MetS^38^. In their study, elevated BP negatively correlated with sleep duration^40^. Nevertheless, our ANCOVA results suggested a positive association between sleep duration and glucose among boys and a positive association between sleep duration and TG among girls, and multiple logistic regression analysis showed that these children exhibited an increased OR for elevated TG. These findings may explain the U-shaped relationship between MetS and sleep duration observed in adulthood. Short sleep duration in the present study was linked to obesity in children and adolescents, which may lead to MetS in adults. A long sleep duration was related to elevated TG, which may be a component of MetS when it persists in adulthood.

In conclusion, this nationally representative, population-based study showed a negative correlation between sleep duration and BMI SDS and WC SDS in boys and a negative correlation between sleep duration and BMI SDS girls using covariance analysis after adjustment for possible confounding factors based on an analysis of sex- and age-specific sleep-duration groups. Children and adolescents in the very short sleep-duration group exhibited an increased OR for overweight/obesity, obesity, and elevated WC and had a lower OR for elevated TG after adjustment for possible confounders using multiple logistic regression analysis. Children and adolescents in the long sleep-duration group had an increased OR for elevated TG. In subgroup analyses based on sex- and age-specific sleep-duration groups, girls showed significant relationships between a very short sleep-duration and overweight/obesity, obesity, and elevated TG, whereas boys exhibited a significant association between a very short sleep-duration and overweight/obesity. Our results suggest that shorter sleep durations may be related to overweight/obesity and obesity (especially central obesity) and that longer sleep durations may be associated with elevated TG. However, the nature of these relationships may be sex-dependent.

## Materials and Methods

### Subjects

Data from the Korea National Health and Nutrition Examination Survey (KNHANES) 2007–2015 were analyzed for the current study. The KNHANES, which is a cross-sectional and nationally representative survey, is conducted regularly by the Division of Chronic Disease Surveillance, Korean Centers for Disease Control and Prevention (KCDC)^41^. The KNHANES is composed of a health questionnaire, health examination, and nutritional assessment and uses a stratified and multistage probability sampling design to select household units. Details of the KNHANES have been described previously^42^. A total of 83,097 participants were included in the KNHANES 2007–2015. Of these, 9,753 children and adolescents aged 10–18 years were enrolled in preliminary analyses. Participants who had not completed the anthropometrical and laboratory examination, including height, weight, WC, SBP, DBP, glucose, T-C, HDL-C, TG, and LDL-C, were excluded (*n* = 1,953). Additionally, subjects who had not received the health questionnaire, which included questions on smoking, alcohol consumption, physical activity, household income, residence, and diagnostic and medication status for hypertension, diabetes mellitus, and dyslipidemia, were excluded from this study (*n* = 1,738). Because LDL-C was determined by Friedewald’s equation^43^, participants with TG ≥400 mg/dL were also excluded (*n* = 14). In the end, a total of 6,048 participants (3,218 boys and 2,830 girls) were included in the current analyses. The database is available to the public at the KNHANES website (http://knhanes.cdc.go.kr). The study protocols of KNHANES 2007–2015 were approved by the Institutional Review Boards of the KCDC. Informed consent was provided by all KNHANES subjects.

### Measurements

Anthropometric assessments were performed by a trained expert using standard methods. In brief, height was assessed to the nearest 0.1 cm using Seca 225 (Seca, Hamburg, Germany), and body weight was measured to the nearest 0.1 kg using GL-6000-20 (G-tech, Seoul, Korea). The WC was determined at the midline from the lower rib margin to the iliac crest to the nearest 0.1 cm. Body mass index (BMI) was assessed as the weight/square of height (kg/m^2^). Analyses used the standard deviation scores (SDS) for height, weight, WC and BMI, which were determined using least mean squares (LMS) methods [SDS = (measured value/M)^1/L^/LS] based on the 2007 Korean reference data^44^. The SBP (mmHg) and DBP (mmHg) were assessed three times at the right upper arm using a calibrated sphygmomanometer (Baumanometer Desk model 0320, Baum, NY, USA) and an appropriately sized cuff. The measurements were taken at 2-minute intervals. Then, the mean of the last two values was used as the SBP and DBP for the analysis.

Random blood samples were obtained from the antecubital vein all the year-round after boys and girls in KNHANES had fasted for at least 8 hours. The collected blood samples were immediately processed, refrigerated, and transported to a central laboratory (NeoDin Medical Institute, Seoul, Korea) for analysis within 24 hours. Blood biochemistry tests, including the levels of glucose (mg/dL), T-C (mg/dL), HDL-C (mg/dL), and TG (mg/dL), were determined using a Hitachi 7600 automatic analyzer (Hitachi, Tokyo, Japan). LDL-C (mg/dL) was calculated according to a previously described method (LDL-C = T-C – HDL-C – TG/5)^43^.

### Collection of general characteristics

Smoking, alcohol consumption, physical activity, household income, and residence were included as lifestyle-related parameters in this study. Smokers were defined as boys and girls who smoked five packs of cigarettes or more throughout their lives, and subjects were divided into two groups (smoker vs. nonsmoker). Alcohol drinkers were defined as individuals who consumed two or more alcoholic beverages per month during the previous year, and subjects were divided into two groups (drinker vs. nondrinker). Physical activity was defined as meeting one or more of the following three criteria: (i) intense physical activity for 20 minutes on three or more days per week, (ii) moderate physical activity for 30 minutes on five or more days per week, or (iii) walking for 30 minutes on five or more days per week. Based on engaging in physical activity, subjects were divided into two groups (yes vs. no). Household income was reported in quartiles, and subjects were categorized into two groups (lowest quartile vs. more than or equal to second quartile). The subject’s place of residence was categorized into two groups (urban vs. rural).

Hypertension, diabetes mellitus, and dyslipidemia were also included as parameters for the subjects’ medical history. Hypertension was defined as (i) having SBP and/or DBP at the 95th percentile or higher for sex, age, and height, and/or (ii) currently taking antihypertensive agents; subjects were categorized into two groups based on the presence of hypertension (yes or no). Diabetes mellitus was defined by fulfilling one or more of the following 3 criteria: (i) self-report on a questionnaire (yes or no), (ii) current administration of medication and/or insulin for diabetes mellitus, or (iii) fasting glucose ≥126 mg/dL during the nationwide health survey; subjects were categorized into two groups (yes or no). Dyslipidemia was also assessed by a self-report questionnaire (yes or no) or by the current use of medication for dyslipidemia, and subjects were categorized into two groups (yes or no).

### Definition of sleep duration

**Figure 2:**
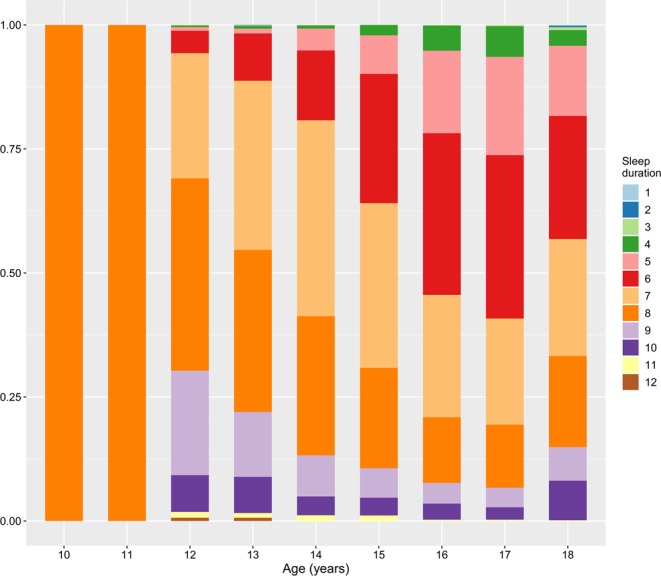
Sleep duration as a function of age in the study participants (*n* = 6,048).

Appropriate and healthy sleep durations vary with age. Sleep duration tends to decrease with age during childhood and adolescence, as described in a previous study^45^. The National Sleep Foundation (NSF) recommends a healthy sleep duration based on age (https://www.sleepfoundation.org/press-release/national-sleep-foundation-recommends-new-sleep-times). In this study, subjects were divided into the following four groups based on age-specific sleep durations, according to NSF recommendations: i) the very short sleep-duration group, which was a sleep duration of <7 hours in subjects aged 10–13 years and <6 hours in adolescents aged 14–18 years; ii) the short sleep-duration group, which was a sleep duration of 7–8 hours in participants aged 10–13 years and 6–7 hours in boys and girls aged 14–18 years; iii) the recommended sleep-duration group, which was sleep duration of 9–11 hours in children aged 10–13 years and 8–10 hours in participants aged 14–18 years; and iv) the long sleep-duration group, which was sleep duration of >11 hours in participants aged 10–13 years and >10 hours in teenagers aged 14–18 years. The recommended sleep-duration group was considered the reference group in this study. The sleep duration of the subjects in this study is shown in Fig. 2.

### Definition of MetS and its components

MetS and its components were defined based on the modified criteria of the National Cholesterol Education Program Adult Treatment Panel III (NCEP ATP III)^46^. Elevated WC was defined as a WC ≥90th percentile according to sex and age. Elevated BP was defined as i) an SBP ≥90th percentile and/or a DBP ≥90th percentile according to sex, age, and height on 2007 Korean growth charts^44^ or ii) the current administration of antihypertensive medicines. Elevated glucose was defined as i) fasting glucose ≥110 mg/dL, ii) subjects who self-reported their disease using a questionnaire comprising yes or no responses, or iii) the current administration of medications to manage T2DM including insulin injections. Elevated TG was defined as i) fasting TG ≥110 mg/dL or ii) the current use of medications for dyslipidemia. Reduced HDL-C was defined as fasting HDL-C <40 mg/dL. MetS was defined as having at least three of the MetS components: (i) elevated WC, (ii) elevated BP, (iii) elevated glucose, (iv) elevated TG, and (v) reduced HDL-C.

### Statistical analyses

All data were analyzed using R version 3.5.1 (The R Foundation for Statistical Computing, Vienna, Austria). Clinical characteristics of the study population were presented according to the four groups of sleep duration separated by sex. Normally distributed continuous variables are presented as the means ± standard deviations (SD), whereas categorical variables are presented as percentages (%). Differences in normally distributed continuous variables and categorical variables were analyzed using one-way analysis of variance (ANOVA) and chi-square (χ^2^) tests according to the sleep duration. The adjusted means ± standard errors (SE) for WC SDS, BMI SDS, SBP, DBP, glucose, T-C, HDL-C, TG, and LDL-C were determined using analysis of covariance (ANCOVA) after adjustment for possible confounding factors based on sex- and age-specific sleep-duration group. WC SDS and BMI SDS were adjusted for sex, age, smoking, alcohol drinking, physical activity, household income, and residence according to the sex-specific sleep-duration groups in model 1, whereas SBP, DBP, glucose, T-C, HDL-C, TG, and LDL-C were adjusted for age, BMI SDS, smoking, alcohol drinking, physical activity, household income, and residence according to the sex-specific sleep-duration groups in model 2. The relationships of sleep duration with overweight/obesity, obesity, MetS components and MetS were evaluated using multivariate logistic regression analysis after adjustment for possible confounders. The adjusted odds ratios (OR) and 95% confidence intervals (95% CI) for overweight/obesity, obesity, MetS components and MetS were determined based on the age-specific sleep-duration groups, with the recommended sleep-duration group serving as a reference. Overweight/obesity and obesity were adjusted for sex, age, smoking, alcohol drinking, physical activity, household income, and residence according to the sleep duration among all participants in model 1. In model 2, MetS and its components were adjusted for sex, age, BMI SDS, smoking, alcohol drinking, physical activity, household income, and residence and analyzed across the sleep-duration groups with all study subjects. To evaluate sex differences in the relationship between sleep duration and overweight/obesity, obesity, and MetS and its components, subgroup analyses were conducted across sex- and age-specific sleep-duration groups. Obesity and overweight/obesity were adjusted for age, smoking, alcohol drinking, physical activity, household income, and residence across sex- and age-specific sleep-duration groups in model 3. In model 4, MetS and its components were adjusted for age, BMI SDS, smoking, alcohol drinking, physical activity, household income, and residence across sex- and age-specific sleep-duration groups. Statistical significance was indicated when the two-tailed *P*-value < 0.05.
